# Preparation of TiO_2_-coated barite composite pigments by the hydrophobic aggregation method and their structure and properties

**DOI:** 10.1038/s41598-017-10620-7

**Published:** 2017-08-30

**Authors:** Sijia Sun, Hao Ding, Hong Zhou

**Affiliations:** Beijing Key Laboratory of Materials Utilization of Nonmetallic Minerals and Solid Wastes, National Laboratory of Mineral Materials, School of Materials Science and Technology, China University of Geosciences, Xueyuan Road, Haidian District, Beijing, 100083 China

## Abstract

We obtained hydrophobic barite (BaSO_4_) and rutile titanium dioxide (TiO_2_) particles (as raw materials) by organic surface modification. Subsequently, TiO_2_-coated barite composite pigments were prepared via the hydrophobic aggregation of heterogeneous particles in a water medium. The pigment properties of the TiO_2_-coated barite composite pigments were characterized and evaluated by determining their hiding power, oil absorption value and whiteness. The optical properties were determined by obtaining their UV-vis diffuse reflectance spectra and using the CIE-L*a*b* method. The morphology and bonding properties were investigated using scanning electron microscopy (SEM), X-ray diffraction (XRD), X-ray photoelectron spectroscopy (XPS), and infrared spectroscopy (IR). The results show the similarity between the composite pigment and pure rutile TiO_2_: when the mass ratio of rutile TiO_2_ in the composite pigment was 60%, the hiding power of the TiO_2_-coated barite composite pigment was 90.81% of that of pure rutile TiO_2_. Moreover, the surfaces of the barite particles were uniformly and firmly coated by TiO_2_, with a hydrophobic association occurring between the hydrophobic carbon chains on the surfaces of barite and TiO_2_ particles.

## Introduction

As a transition metal oxide with excellent properties, TiO_2_ has demonstrated utility in many applications, including solar energy harvesting, photocatalytic hydrogen production^1, 2^ and white pigment. A series of TiO_2_ species with different morphology, structure and properties have been prepared, such as TiO_2_ nanotubes^3, 4^ TiO_2_ nanosheets, and TiO_2_ microparticles. Among these species, TiO_2_ nanotube-based materials have great potential in various applications as photocatalysts^5, 6^ and sensors because of their outstanding optical^7, 8^ and electrical properties. Additionally, in the pigment field, TiO_2_ microparticles have advantages for certain applications because of their appropriate particle size, which locates them within the best range for scattering visible light. Titanium dioxide pigment is a functional powder material consisting of the TiO_2_ crystal phase with a particle size of 200–300 nm. The high refractive index of TiO_2_ (2.7 of rutile and 2.3 of anatase) endows it with excellent properties, such as a strong hiding power, a high tinctorial strength, a high glossiness, and a strong whiteness. Therefore, titanium dioxide has become the best white pigment and is applied in numerous fields, for example, in paints, plastics, paper, inks and rubber products^9, 10^. However, there are several problems with the application of TiO_2_, such as particle aggregation and poor compatibility with organic matrices. Because of these hindrances, the pigment properties of TiO_2_ are often exerted ineffectively, causing a reduction in its efficiency and an increase in the amount used. Consequently, concerns regarding the environment and increased resource use during the production of TiO_2_ have arisen^11, 12^. To improve the dispersion of TiO_2_ and the compatibility of TiO_2_ with organic matrices, researchers have focused on developing new inorganic pigments comprising inorganic particles uniformly and firmly coated with TiO_2_^13^. In these research, many inorganic non-metallic minerals have been used as substrates, including calcined kaolin^14^, sericite^15^, and silica^16^. Methods such as homogeneous hydrolysis^17^ and mechanochemistry have also been applied to the preparation of TiO_2_-coated inorganic composite particles.

Barite is an inorganic non-metallic mineral with a chemical composition of BaSO_4_. Traditionally, the barite industry mainly produces primary products of low-added value, including petroleum, weighting material for drilling mud in natural gas operations, and barium-containing chemical products^18^. Recently, a series of studies on new functional barite materials have been conducted to exploit the excellent optical properties, good dispersibility, chemical stability, and anti-radiation properties of fine barite particles and to explore synergistic effects^19^. Barite has a strong whiteness, a low oil absorption and a similar density to TiO_2_. Therefore, composite pigments with a similar density to TiO_2_ can be prepared using barite and TiO_2_ as raw materials. In this way, the mixed homogenization of a composite pigment and a matrix material can be improved, resulting in an improvement in production. Zhou^20^ has prepared barite-TiO_2_ composite particles by hydrolysing TiOSO_4_ on the surfaces of barite particles. Wang^21^ and Zhou^22^ have separately used the mechanochemistry method to prepare anatase and rutile TiO_2_-coated barite composite pigments with pigment properties are similar to those of TiO_2_.

By contrast, to prepare TiO_2_-coated barite composite pigments via the hydrolysis of TiOSO_4_^20^ the precipitation product is amorphous TiO_2_, which should be calcined at 800–900 °C for conversion to crystalline TiO_2_. Inevitably, the calcination consumes a large amount of energy, and converting the TiO_2_•nH_2_O on the surface of barite into the rutile single-crystal phase is difficult. The mechanochemistry method^21, 22^ also consumes considerable energy because the composite pigments are prepared using a grinding mill with high power. Meanwhile, TiO_2_-coated barite composite pigments prepared by the two aforementioned methods have a hydrophilic surface, leading to their poor compatibility with organic matrices and a reduction in their usage efficiency. For the above reasons, a hydrophobic aggregation method has been used in this study in order to obtain uniformly and firmly TiO_2_-coated barite composite pigments with a hydrophobic surface while consuming less energy.

Hydrophobic aggregation refers to the aggregation of fine particles that attract each other because of their hydrophobic surfaces^23^. The attraction occurs at a particle distance ranging from 2 to 25 nm in a water medium, and the attraction strength is 10–100 times stronger than that of the electrical double layer force and Van der Waals force^24^. In particular, when the hydrophobic surfaces of particles are induced by surface modification, a strong aggregation occurs among the particles because of the association between the hydrocarbon chains on the surfaces. The abovementioned condition results in the particles combining firmly. The hydrophobic aggregation phenomenon of particles was first observed in the mineral flotation process, which has been improved and applied constantly. However, the existing studies mainly focus on the hydrophobic aggregation of homogeneous mineral particles in raw ore. For example, Song^25, 26^ has studied the hydrophobic aggregation and separation of fine rutile, haematite and hydrophobic coal particles. Furthermore, research regarding the hydrophobic aggregation and flotation of galena, sphalerite, rhodochrosite and other fine-grained minerals has been reported^27^. By contrast, there are fewer studies on the hydrophobic aggregation of heterogeneous particles, especially for the preparation of composite pigments through the hydrophobic aggregation of TiO_2_ and mineral particles. Because the hydrophobic aggregation of TiO_2_ and mineral particles needs to be based on the selective recognition among particles, performing this work is a significant challenge. In this study, TiO_2_-coated barite composite pigments were prepared via the hydrophobic aggregation of barite and TiO_2_ particles in a water medium. Meanwhile, their morphology, structure, and bonding properties were studied. Their pigment properties were also characterized and evaluated.

## Experimental

### Materials

**Figure 1:**
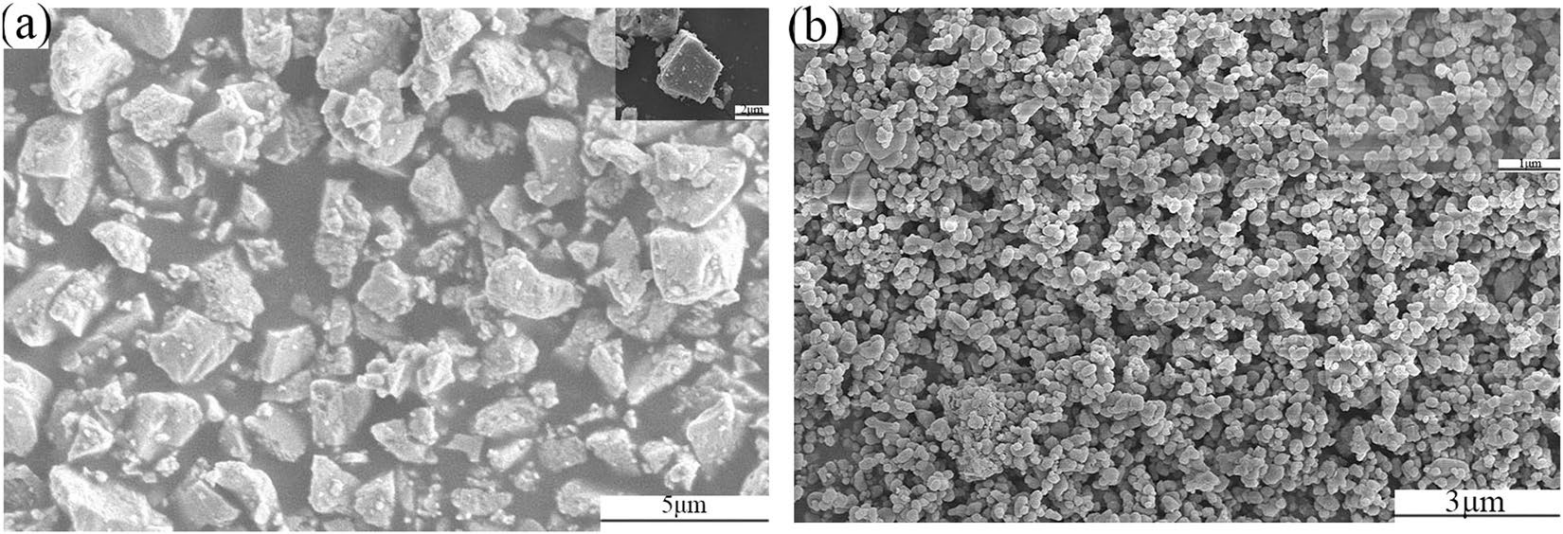
SEM images of (**a**) barite raw material and (**b**) rutile TiO_2_ raw material.

The barite raw material used as the substrate in this study was produced in Hubei Province, China, and had a purity of 100%, a whiteness of 89.40%, a hiding power of 155.00 g/m^2^ and an oil absorption of 11.40 g/100 g. Figure 1(a) shows the morphology of the barite raw material, as determined by scanning electron microscope (SEM), wherein the barite particles were well dispersed and exhibited plate- and lump-like shapes. The particles possessed a uniform size distribution.

The rutile TiO_2_ raw material was produced in Henan Province, China, and had a whiteness of 94.40%, a hiding power of 10.97 g/m^2^, and an oil absorption of 24.26 g/100 g. Figure 1(b) shows the granular rutile TiO_2_ with an average particle size of 300 nm.

Analytical-grade sodium stearate (CH_3_(CH_2_)_16_COONa), sulfuric acid (H_2_SO_4_) and sodium hydroxide (NaOH) were used in the experiments. Meanwhile, we used chemically pure linseed oil and distilled water.

### Preparation method

**Figure 2:**
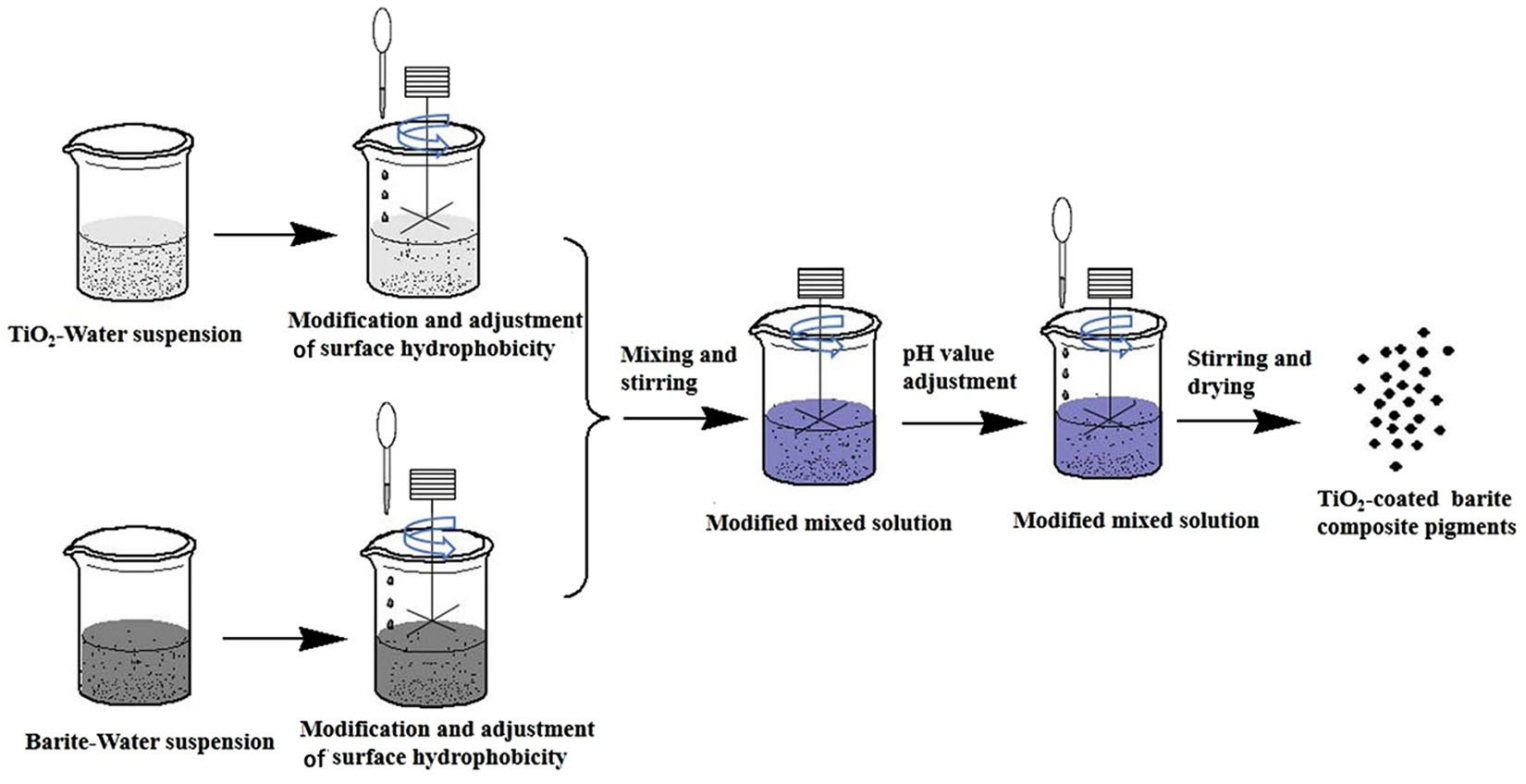
Flowchart of the preparation of the TiO_2_-coated barite composite pigments.

The TiO_2_-coated barite composite pigments were prepared via the hydrophobic aggregation method, which is illustrated in the flowchart in Fig. 2. The modified TiO_2_ slurry (MTS) was obtained through the following way: rutile TiO_2_ powder was dispersed in water, mixed with 3% mass ratio modifier (CH_3_(CH_2_)_16_COONa) and stirred for approximately 45 min at 60 °C. And for comparison, the unmodified TiO_2_ slurry (UTS) was prepared via the same process without the addition of modifier. Similarly, the modified barite slurry (MBS) was prepared: barite powder was dispersed in water, mixed with 1% mass ratio modifier (CH_3_(CH_2_)_16_COONa) and stirred for approximate 45 min at 70 °C, and the unmodified barite slurry (UBS) was also prepared via the same way without the addition of modifier. Afterwards, the composite process was conducted: the MBS was mixed with different mass ratios of the MTS. After stirring for 60 min at a pH value of 9, we obtained a barite-TiO_2_ slurry. Finally, the barite-TiO_2_ slurry was dried at 60 °C, and then the TiO_2_-coated barite composite pigments were prepared.

As a comparison, composite particles fabricated using UBS and UTS as raw materials were also prepared (discussed in 3.4.1). For Sample 1, the composite materials were prepared using UBS and UTS as the raw materials, the Sample 2 used MBS and UTS as raw materials, and the Sample 3 used UBS and MTS as raw materials. For all the three samples, the mass ratio of TiO_2_ to barite was 6:4, and the composite process was carried out through the way described in the previous paragraph. Subsequently, all the prepared slurries were dried at 60 °C to obtain the composite particles.

### Characterization

#### Pigment properties

The pigment properties of the TiO_2_-coated barite composite pigments were evaluated by determining the oil absorption value, hiding power, relative hiding power and whiteness. The test methods were as follows.

Oil absorption is an important index of pigment properties, this value refers to the minimum amount of varnish (linseed oil) that can wet 100 g of the pigment completely. The oil absorption of a pigment can be tested basing on the China National Standard GB/T5211.15-2014.

Hiding power is another important and insightful index of pigment properties. It refers to the minimum amount of pigment that can completely cover a black and white checkerboard. The hiding power of a pigment can be tested using the National Industry Standards HG/T3851-2006 (the test method of pigment hiding power).

The relative hiding power (R) indicates the hiding property ratio of the composite pigment to a pure TiO_2_ pigment according to the definition of pigment hiding power. The R value can be calculated using Equation (1)

1 $${\rm{R}}=({{\rm{H}}}_{{\rm{T}}}/{{\rm{H}}}_{{\rm{CT}}})\times 100 \% $$

where H_T_ (g/m^2^) and H_CT_ (g/m^2^) are the hiding powers of TiO_2_ and the TiO_2_–coated barite composite pigments, respectively.

The value of ΔR calculated by Equation (2) represents the magnitude of the increase in the hiding power of TiO_2_ upon forming its composite with barite.

2 $${\rm{\Delta }}{\rm{R}}={\rm{R}}-{{\rm{R}}}_{{\rm{0}}}$$

where R_0_ is the mass ratio of TiO_2_ to the composite pigment.

The whiteness was tested using a whiteness meter (SBDY-1, Shanghai Yuet Feng Instrument Co. Ltd., China).

#### Optical properties

The optical properties of the TiO_2_-coated barite composite pigments were characterized by acquiring their UV-vis diffuse reflectance spectra and using the CIE-L*a*b* method. The UV-vis spectra of the prepared composite pigments were obtained between 200 and 800 nm on a TU-1901 double beam spectrophotometer. The L*a*b* parameters of representative specimens were measured using a portable integrating sphere spectrophotometer (X-Rite Sp60, X-Rite (Shanghai) International Trade Co., Ltd., China). The CIE-L*a*b* colourimetric method recommended by the CIE (Commission Internationale de l’Éclairage) was followed. In this colour system, L* is the colour lightness (L* = 0 for black and 100 for white), a* is the green (−)/red (+) axis, and b* is the blue (−)/yellow (+) axis^28^.

#### Morphology and structure

The hydrophobic properties of the particles were evaluated based on the wet contact angle obtained by contact angle measurement (JC2000D, Shanghai Zhongchen Digital Technic Apparatus Co. Ltd., China). First, the powder samples were pressed into a thin sheet with a smooth surface by a tablet pressing machine. Next, the contact angle of the samples was measured three times. Then, the results were averaged. The images of TiO_2_-coated barite particles dispersed in an organic medium were obtained by an image analyser (BT-1600, Bettersize Instruments Ltd., China)

We observed the morphology of barite, rutile TiO_2_, and TiO_2_-coated barite composite pigments by SEM (S-3500N, HITACHI, Japan). The surface functional groups were examined by infrared spectroscopy (Spectrum 100, PerkinElmer Instruments (Shanghai) Co. Ltd., China) using KBr as the medium. Further analysis was carried out using X-ray photoelectron spectroscopy (XPS, Escalab 250xi, Thermo Fisher Scientific USA) and X-ray diffraction (D/MAX2000, Rigaku Corporation, Japan).

## Results and Discussion

### Pigment properties of TiO_2_-coated barite composite pigments

Barite and rutile TiO_2_ particles were modified using sodium stearate. Based on the induced surface hydrophobicity of the modified particles (the wetting contact angles of barite and TiO_2_ particles are 128.5° and 114.2°, respectively), TiO_2_-coated barite composite pigments with different TiO_2_ mass ratios were prepared via the hydrophobic aggregation method. Table 1 shows the pigment properties of the TiO_2_-coated barite composite pigments and raw materials.

**Table 1:** Pigment properties of the TiO_2_-coated barite composite particles.

Mass ratio of rutile TiO_2_ (R_0_) /%	Contact angle/°	Oil absorption /(g/100 g)	Whiteness/%	Hiding power/(g/m^2^)	Relative hiding Power (R)/%	ΔR (R-R_0_) /%
0 (barite)	22.50	11.40	89.40	155.00	—	—
20	95.80	12.20	90.12	22.85	48.01	28.01
40	110.20	12.56	90.97	15.18	72.27	32.27
50	112.50	12.85	91.35	13.16	83.36	33.36
60	115.60	14.48	91.80	12.08	90.81	30.81
80	109.40	16.79	92.39	11.15	98.38	18.38
100(TiO_2_)	33.20	24.26	94.40	10.97	100.00	—

The results show that the hiding power of the barite raw material is 155.00 g/m^2^, which indicates no hiding properties. However, the hiding power clearly improved after the barite particles were coated by TiO_2_ particles and increased as the TiO_2_ mass ratio increased. When the TiO_2_ mass ratio increased to 60%, the hiding power and relative hiding power (R) of the TiO_2_-coated barite composite pigments were 12.08 g/m^2^ and 90.81%, respectively, nearly reaching the levels observed for pure rutile TiO_2_, for which the R value exceeds 30.81% (ΔR) compared with the mass ratio of TiO_2_ (R_0_). In addition, the TiO_2_-coated barite composite pigments had a lower oil absorption value than that of TiO_2_ and a similar whiteness to TiO_2_. Additionally, the contact angle of the composite particles was more than 100°. The above results show that the TiO_2_-coated barite composite pigments possessed pigment properties that were completely different from those of barite but similar to those of TiO_2_. Furthermore, the composite pigments exhibited hydrophobicity. It can be concluded that the barite particles were uniformly and firmly coated by TiO_2_ particles.

**Figure 3:**
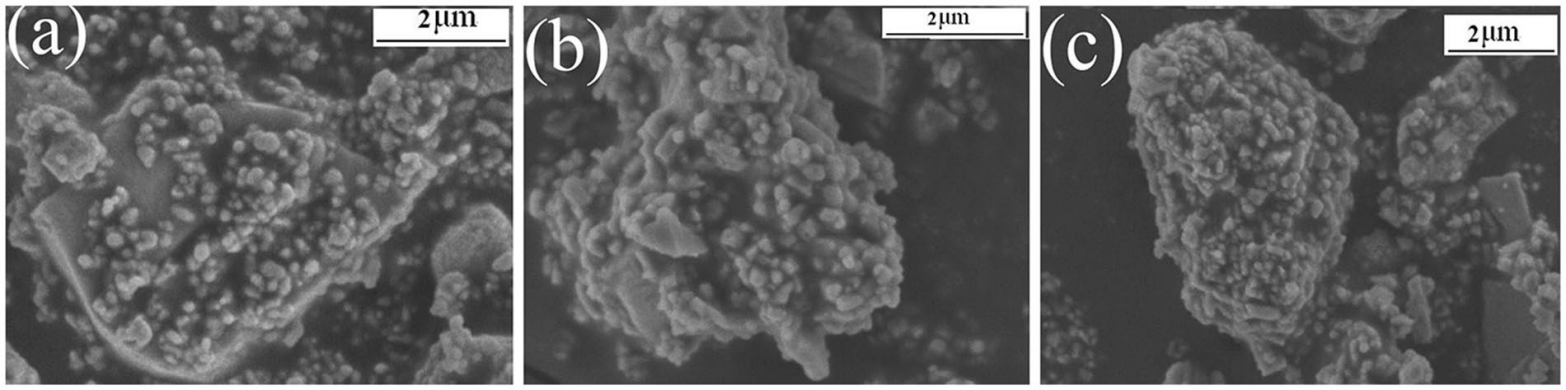
SEM images of the barite-TiO_2_ composites with different TiO_2_ mass ratios(**a**) 20%, (**b**) 40%, and (**c**) 60%.

Figure 3 shows the morphology of the TiO_2_-coated barite composite pigments with different TiO_2_ mass ratios. Figure 3a,b and c show that the number of TiO_2_ particles and the coated area of the barite surface increased remarkably as the TiO_2_ mass ratio increased from 20% to 60%, giving a uniform coating. Particularly, when the TiO_2_ mass ratio increased to 60%, the surfaces of the barite particles were nearly completely covered by the TiO_2_ particles. The results in Table 2 show the atomic percentages of different samples obtained from XPS analysis. Clearly, as the mass fraction of TiO_2_ increases, the percentage of Ti atoms on the surface of the barite particles increased, whereas the number of Ba atoms decreased. Meanwhile, the value of Ti/Ba increased as the TiO_2_ mass fraction increased. The above results show that the TiO_2_ coated on the barite surface becomes more compact as the TiO_2_ content increases. The SEM and XPS results are consistent with the results in Table 1.

**Table 2:** Atomic percentage of the samples according to XPS analysis.

mass fraction of TiO_2_/%	Atomic percentage/%						Ti/Ba
	C	S	Ti	O	Ba	Sum	
20	18.21	11.01	7.07	53.32	10.39	100.00	0.68
40	15.00	8.59	10.63	56.96	8.82	100.00	1.21
60	16.64	6.36	13.99	56.77	6.25	100.00	2.24

### Optical properties of TiO_2_-coated barite composite particles

There are several methods for measuring the colour of pigments, and the CIE-L*a*b* values were used to specify and compare the colour of the TiO_2_-coated barite composite pigments with different TiO_2_ mass ratios. Table 3 shows the results.

**Table 3:** Colourimetric coordinates (L*, a*, and b*) and total colour difference (∆E) between the TiO_2_-coated barite composite pigments and rutile TiO_2_.

Samples	TiO_2_ mass ratio (%)	Colour coordinates			∆E
		L*	a*	b*	
Barite	0	93.44	2.28	3.35	—
Rutile TiO_2_	100	97.04	−0.44	0.51	—
Barite-TiO_2_	20	95.34	0.30	1.05	1.93
Barite-TiO_2_	40	96.16	0.47	0.98	1.35
Barite-TiO_2_	60	97.34	0.13	0.66	0.66

The L* value of the TiO_2_-coated barite composite pigment gradually increased with the TiO_2_ mass ratio until it was close to that of rutile TiO_2_. This observation indicates that the lightness of the composite pigments improved because of the coating of TiO_2_ particles on the barite particles surface. Similarly, the a* and b* values of the TiO_2_-coated barite composite particles are also close to those of rutile TiO_2_ at increased TiO_2_ mass ratios. Meanwhile, the total colour difference (∆E) between the TiO_2_-coated barite composite pigments and rutile TiO_2_, which was used to compare the colour of two samples, ranges from 0.66 to 1.93. The ∆E value decreased as the TiO_2_ mass ratio increased. These results indicate that the TiO_2_-coated barite composite pigments display a similar visual colour to rutile TiO_2_ because of the coating of TiO_2_ particles on the barite particle surface.

**Figure 4:**
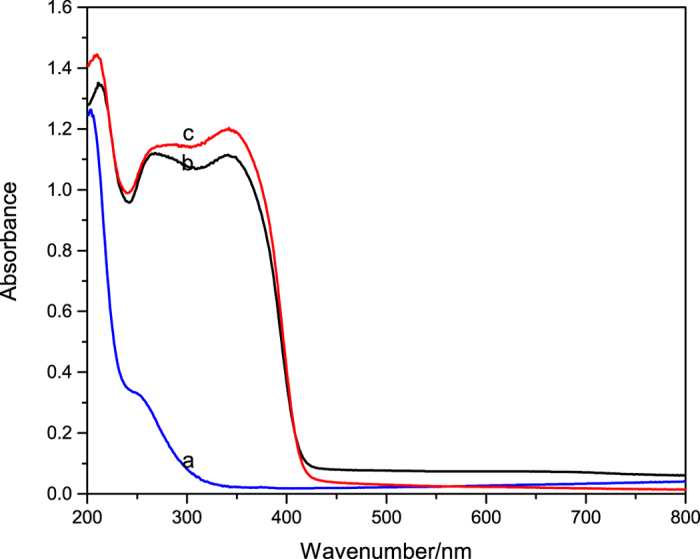
UV-vis absorption spectra of (**a**) barite, (**b**) the TiO_2_-coated barite composite pigments, and (**c**) rutile TiO_2_.

The UV-vis absorption spectra of TiO_2_-coated barite composite pigment (TiO_2_ mass ratio was 60%), barite and TiO_2_ were obtained and compared (See Fig. 4). There was less light absorbed by barite between 300 and 400 nm, whereas TiO_2_ absorbed light of wavelengths below 400 nm. The TiO_2_-coated barite composite pigments exhibited light absorption in a wavelength range from 200 to 400 nm. These pigments also show high light absorption at wavelengths of 300–400 nm, in complete contrast to barite but similar to TiO_2_. The results indicate that the TiO_2_-coated barite composite pigment has equivalent UV absorption and anti-UV properties, confirming that the barite is coated by TiO_2_ particles with similar optical properties to TiO_2_.

### Compatibility with an organic matrix

**Figure 5:**
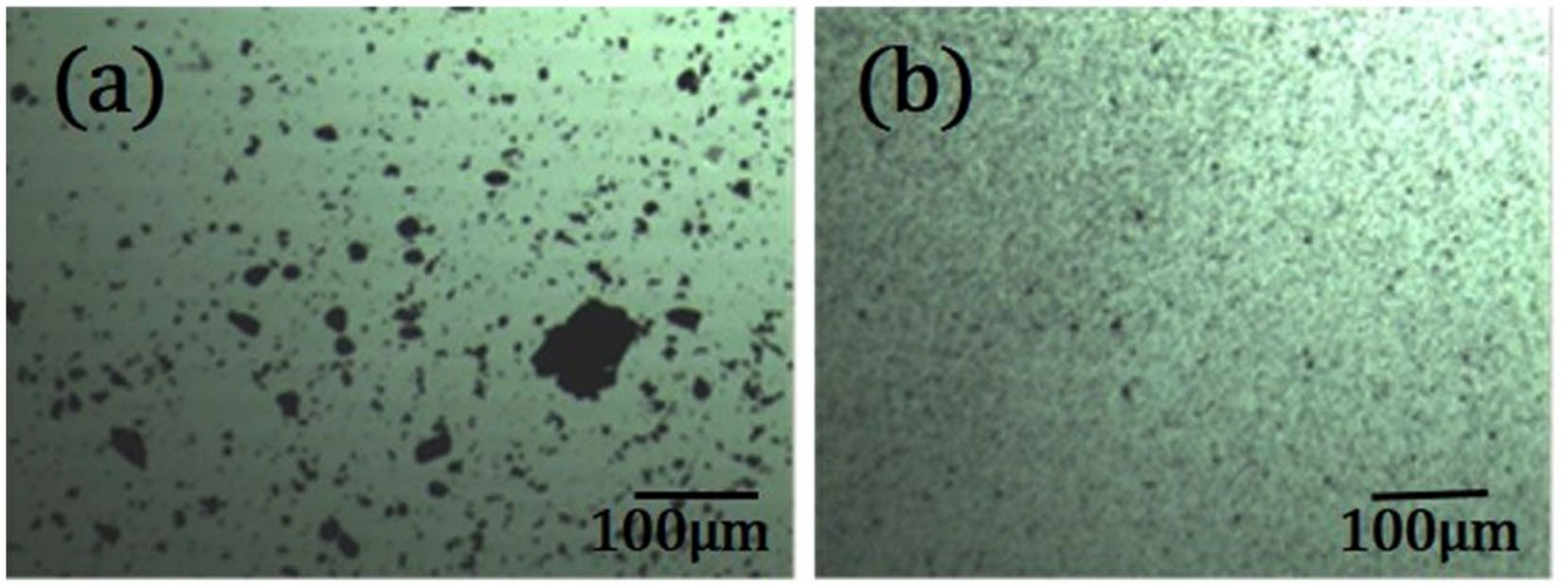
Images of TiO_2_-coated barite composite particles dispersed in a kerosene medium.The TiO_2_-coated barite composite particles were prepared by (**a**) the mechanochemical method and (**b**) the hydrophobic aggregation method.

To investigate the compatibility of the TiO_2_-coated barite composite particles with an organic matrix, a dispersion experiment was performed. In this experiment, 1 g of composite particles was dispersed in 100 mL of kerosene to prepare a suspension, which was stirred for 30 min at a speed of 800 rpm. After stirring, a small amount of the suspension was taken to prepare samples. Images of the composite pigments in the kerosene medium are shown as Fig. 5. There are two types of particles in this experiment: the TiO_2_-coated barite composite particles in Fig. 5(a), which were prepared by the mechanochemical method^21, 22^ and the particles in Fig. 5(b), which were prepared by the hydrophobic aggregation method.

Clearly, the particle clusters in Fig. 5(a) are significantly larger than those in Fig. 5(b). In the kerosene medium, the TiO_2_-coated barite composite particles prepared by the mechanochemical method agglomerate to form big aggregate particles with a size of 20–50 μm, which reflects their poor compatibility with the organic matrix. However, the particles observed in Fig. 5(b) possess a smaller particle size and exhibit good dispersion, indicating that the TiO_2_-coated barite composite particles with a hydrophobic surface have good compatibility with the organic matrix.

### Bonding properties of barite and TiO_2_ particles

#### Effect of barite and TiO_2_ particles’ surface hydrophobicity

To investigate the effect of surface hydrophobicity and the carbon chain of the modifier on TiO_2_ and barite particles and to further elucidate the bonding mechanism, TiO_2_-coated barite composite pigments (the TiO_2_ mass ratio was 60%) were prepared under the following conditions: The barite raw material had a contact angle of 22.5°, the modified barite material had a contact angle of 128.5°, the TiO_2_ raw material had a contact angle of 33.2°, and the modified TiO_2_ had a contact angle of 114.2°. The preparation details of the four samples are described in experiment section, Table 4 shows the pigment properties.

**Table 4:** Pigment properties of the rutile TiO_2_-coated barite composite particles.

Samples	Mass ratio of TiO_2_ (R_0_)/%	Oil absorption/(g/100 g)	Hiding power/(g/m^2^)	Relative hiding Power (R)/%	ΔR (R-R_0_)/%
1	60	11.40	16.61	66.04	6.04
2	60	11.20	15.37	71.37	11.37
3	60	12.56	14.82	74.02	14.02
4	60	14.48	12.08	90.81	30.81

Sample 1 Barite + TiO_2_, Sample 2 Modified barite + TiO_2_, Sample 3 Barite + Modified TiO_2_, and Sample 4 Modified barite + Modified TiO_2_.

Among the four samples, Sample 4 exhibited the best pigment properties with a hiding power and a relative hiding power (R) of 12.08 g/m^2^ and 90.81%, respectively. By contrast, the other three samples exhibited poor hiding properties with ∆R values ranging only from 6.04 to 14.04%, this result indicates the poor compositing of these samples. Based on these findings, it can be concluded that the composite performs effectively only when both the barite and TiO_2_ surfaces exhibit hydrophobicity. Because the hydrophobicity of TiO_2_ and barite particles is obtained via modification with sodium stearate, the organic carbon chains of the modifier on the particle surface play an important part in the composite process. These observations demonstrate the hydrophobic interaction of barite and TiO_2_ particles.

**Figure 6:**
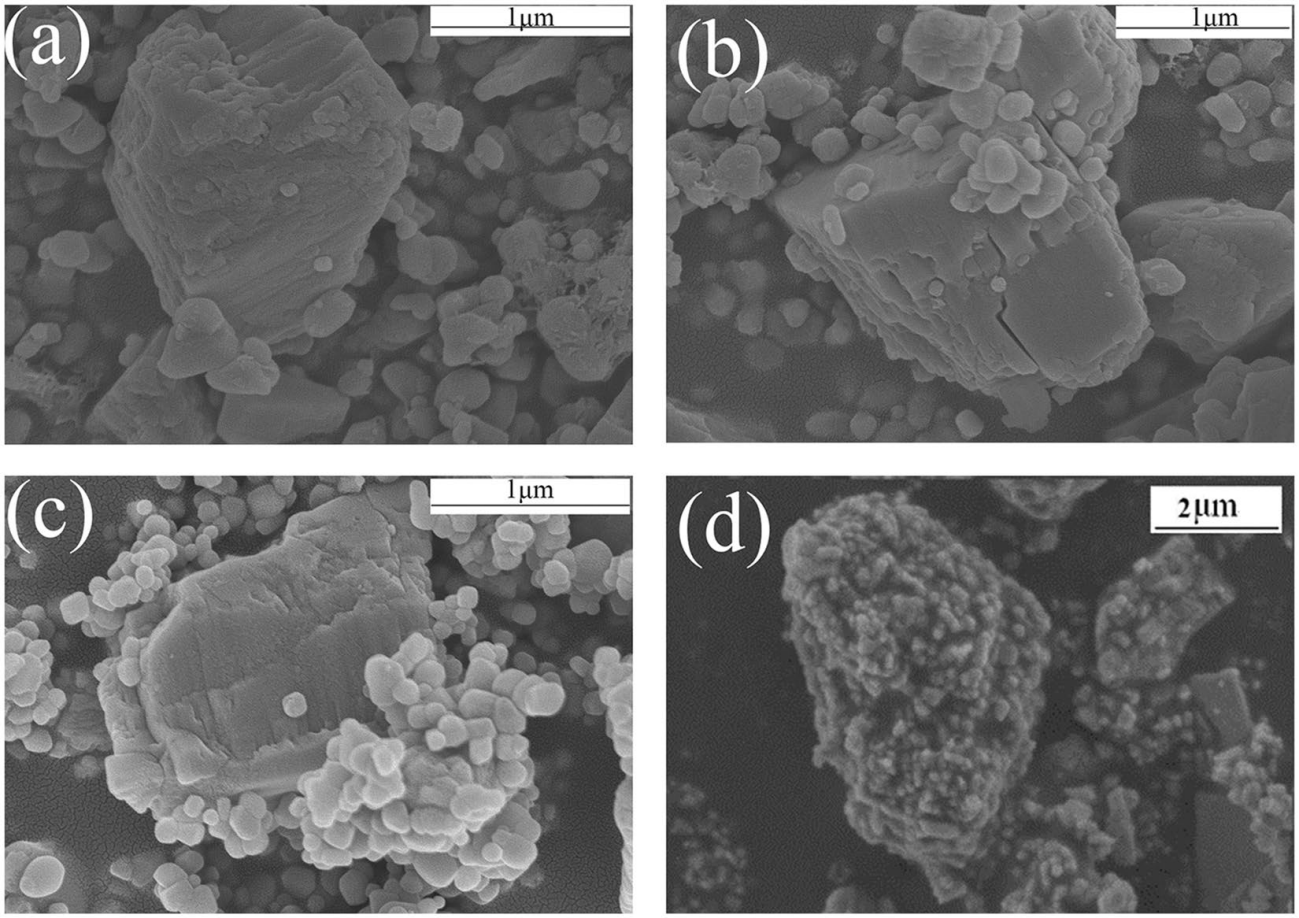
SEM images of (**a**) Sample 1, (**b**) Sample 2, (**c**) Sample 3 and (**d**) Sample 4.

**Figure 7:**
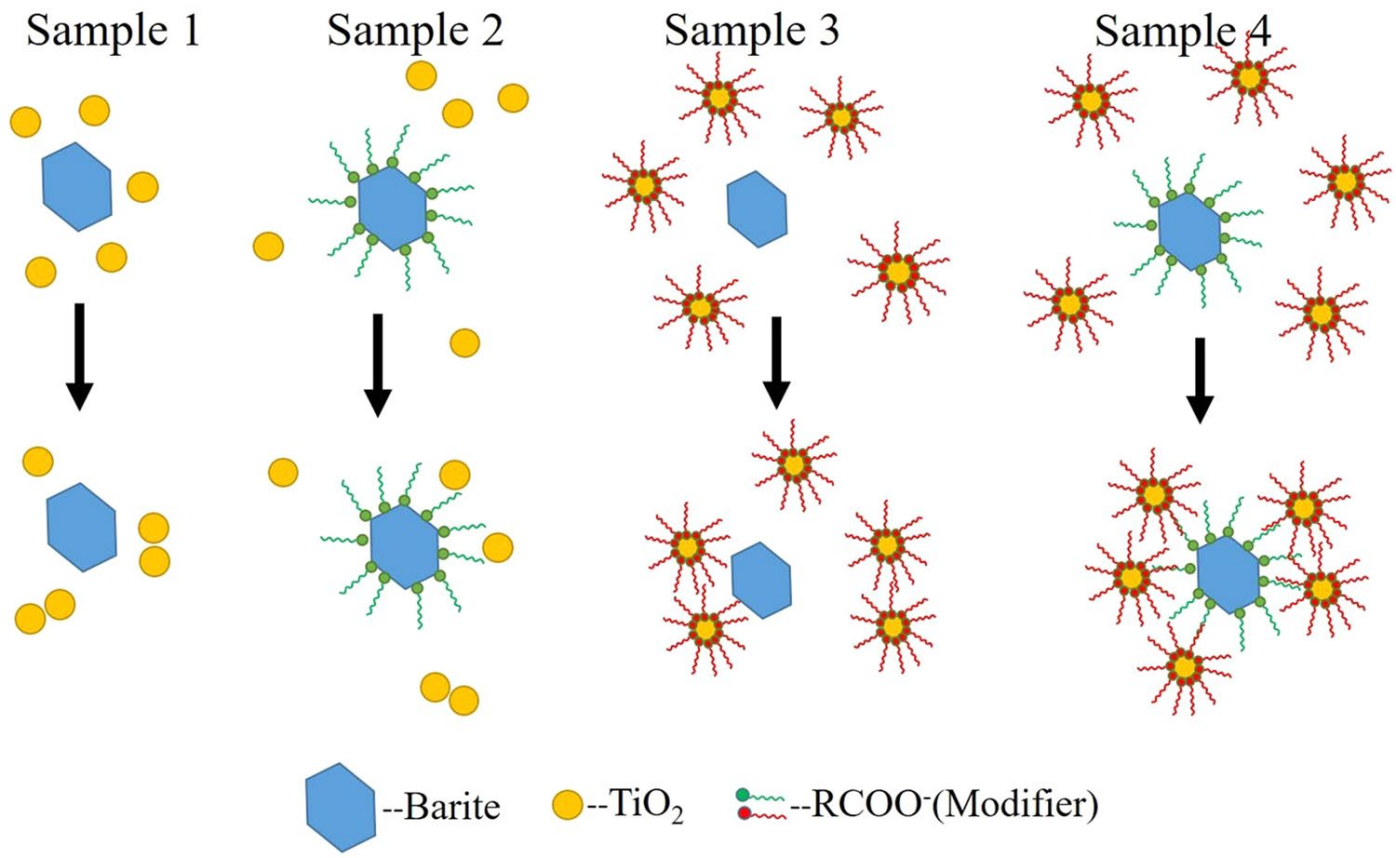
Composite model of barite and TiO_2_ particles with different surface hydrophobicities.

Figure 6 shows SEM images of the four samples. The clear view of the exposed surface of barite in Samples 1, 2, and 3 indicate that the barite particles in these samples have not been well coated by the TiO_2_ particles. By contrast, the barite-TiO_2_ composite particles in Sample 4 show an excellent coating morphology, where the barite particles are thoroughly coated by the TiO_2_ particles. Figure 7 shows the composite model. The microstructure of the composite particles in the four samples can be used to explain the results in Table 4.

#### Bonding strength analysis of barite and TiO_2_ particles

**Figure 8:**
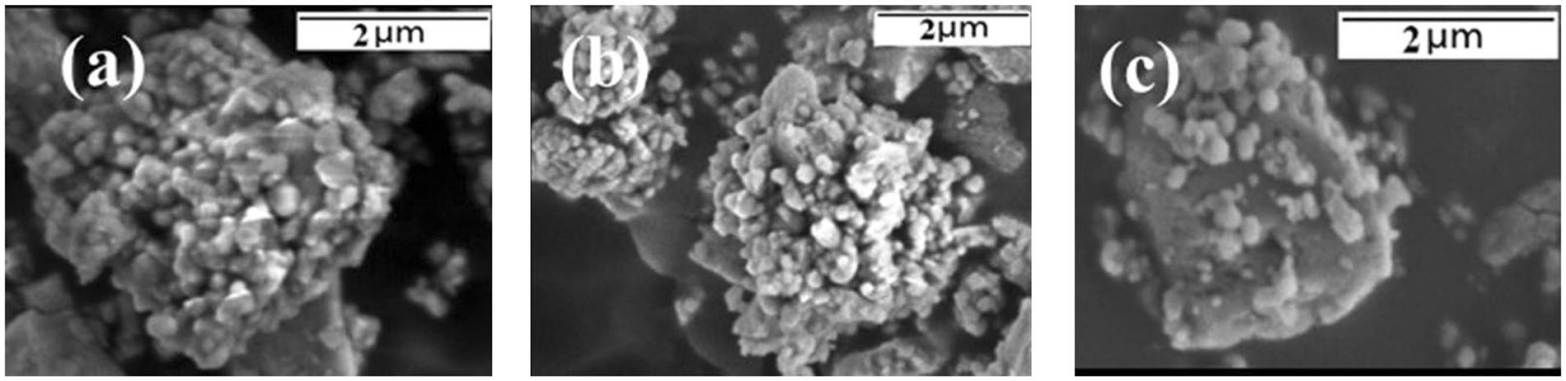
SEM images of barite-TiO_2_ composite particles after sonication:(**a**) 100 W, 10 min; (**b**) 400 W, 10 min; and (**c**) 800 W, 10 min.

The bonding strength between barite and TiO_2_ particles is undoubtedly another key factor influencing the composite pigment properties. To evaluate the bonding strength between the barite and TiO_2_ particles, an experiment based on the ultrasonic treatment of the TiO_2_-coated barite composite pigments was carried out. First, the as-prepared TiO_2_-coated barite composite pigments were mixed with ethanol-water (the mass ratio of ethanol to water was 1:4) to prepare a suspension, and then the suspension was ultrasonicated for 10 min at different powers with an ultrasonic oscillator. Finally, the samples were obtained after drying. Figure 8 shows the SEM images of the samples.

The barite particles were still coated by TiO_2_ particles after the TiO_2_-coated barite composite pigments were ultrasonicated at powers of 100 and 400 W. When the power increased to 800 W, only a small amount of the TiO_2_ particles were removed from the barite particle surface. These results indicate that the bonding between barite and TiO_2_ particles is strong enough, as the energy of ultrasonic vibration is substantially stronger than the Van der Waals forces of barite-TiO_2_ particles^21^. This phenomenon can account for the similarity in pigment properties between the composite pigment and TiO_2_. The aforementioned results also indicate that the bonding between TiO_2_ and barite in the TiO_2_-coated barite composite pigments is chemical or another physical combination with relatively large bonding energy.

#### XRD analysis

**Figure 9:**
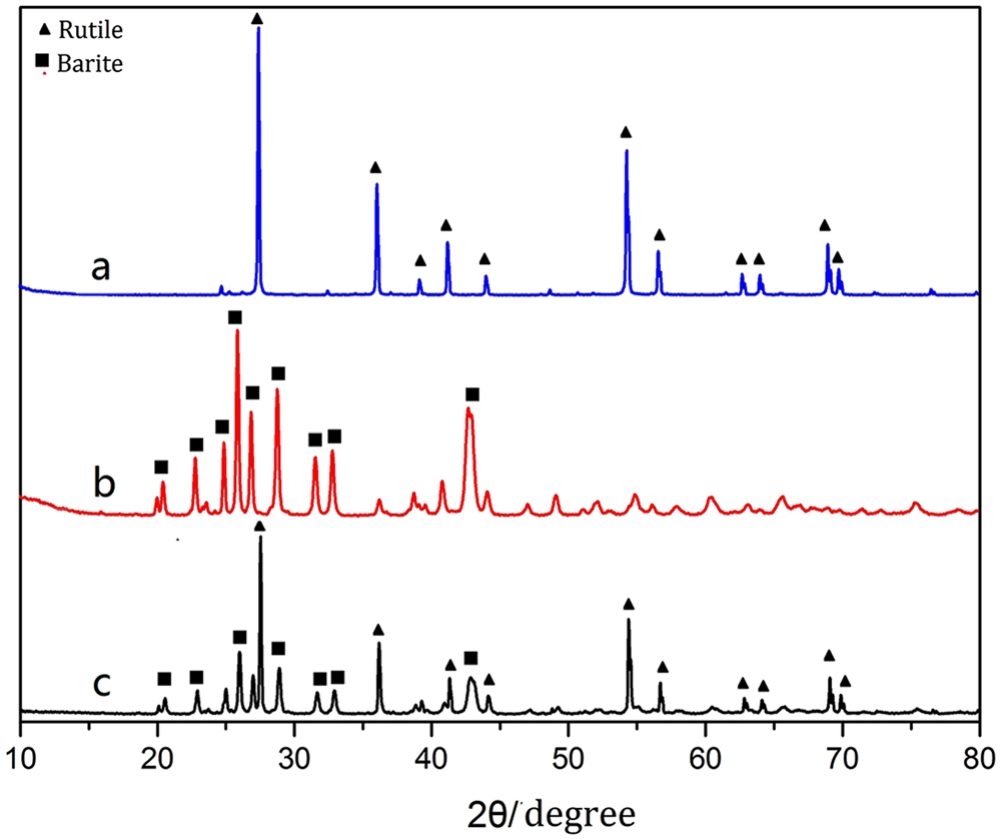
XRD patterns of (**a**) TiO_2_, (**b**) barite and (**c**) the TiO_2_-coated barite composite pigments.

The XRD patterns of TiO_2_, barite and TiO_2_-coated barite composite pigments (see Fig. 9) show that the raw materials used in this study comprise pure barite (JCPDS 24–1035) and rutile crystal phase (JCPDS 21–1276). Only the diffraction peaks of barite and rutile appear at Fig. 9c, indicating that there is no new phase produced in the process of preparing the composite particles. Meanwhile, the barite and TiO_2_ raw materials remain firmly in their complete crystal phases. Therefore, it can be inferred that the barite and TiO_2_ particles form a mixed phase rather than undergoing a chemical reaction during the preparation process.

#### XPS analysis

**Figure 10:**
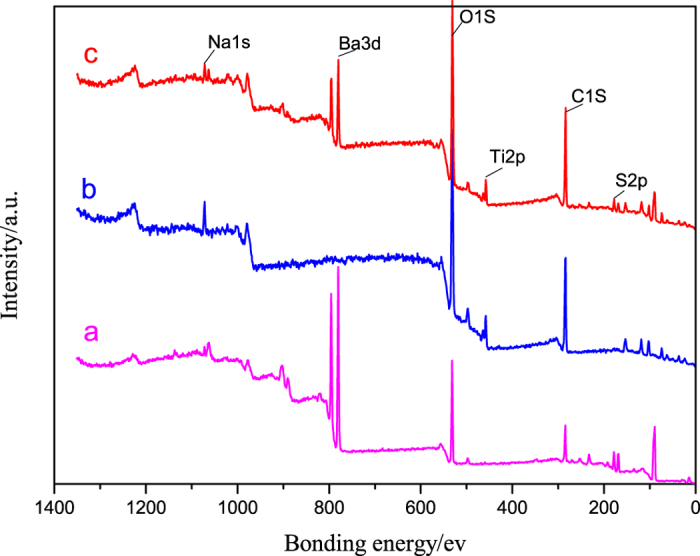
XPS spectra of (**a**) modified barite, (**b**) modified TiO_2_, and (**c**) the TiO_2_-coated barite composite pigments.

The XPS analysis was carried out to prove the presence of TiO_2_ particles in the TiO_2_-coated barite composite pigment (the TiO_2_ mass ratio is 60%), and also to elucidate the bonding mechanism. Figure 10 shows the wide-scan XPS spectra of the modified barite, modified rutile TiO_2_, and TiO_2_-coated composite pigments.

The spectrum of the modified barite contains Ba 3d, S 2p and O 1s peaks, whereas the spectrum of modified TiO_2_ shows Ti 2p and O 1s peaks, consistent with the composition of the raw materials. In contrast to the modified barite, the spectrum of the TiO_2_-coated barite composite pigment contains the Ti 2p peaks, suggesting that the barite surface was indeed coated with rutile TiO_2_.

**Figure 11:**
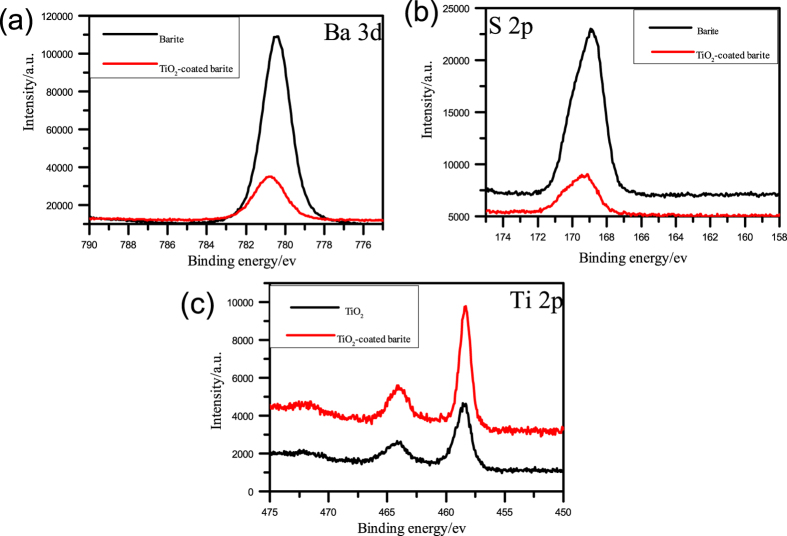
XPS narrow-scan spectra of (**a**) Ba 3d, (**b**) S 2p, and (**c**) Ti 2p.

Figure 11 shows the narrow-scan spectra of Ba 3d, S 2p, and Ti 2p in the modified barite, rutile TiO_2_ and composite particles. In Fig. 11(a), the Ba 3d peaks appear at 780.40 eV and 780.75 eV, with the two peaks nearly overlapping. This finding indicates that the chemical state of barite was not changed during the compositing. The S 2p peaks in Fig. 11(b) appear at 169.10 eV and 168.95 eV and are assigned to SO_4_^2−^. This result indicates that SO_4_^2−^ is not involved in any chemical reaction^29^. Meanwhile, the peaks observed at 458.55 and 458.40 eV in the XPS spectrum of Ti 2p (see Fig. 11(c)) represent the Ti 2p_3/2_ species of Ti^4+^ in TiO_2_^30, 31^, confirming that the chemical state of TiO_2_ was not changed. The abovementioned results show that no chemical reaction occurred between the barite and TiO_2_ particles.

#### IR analysis

**Figure 12:**
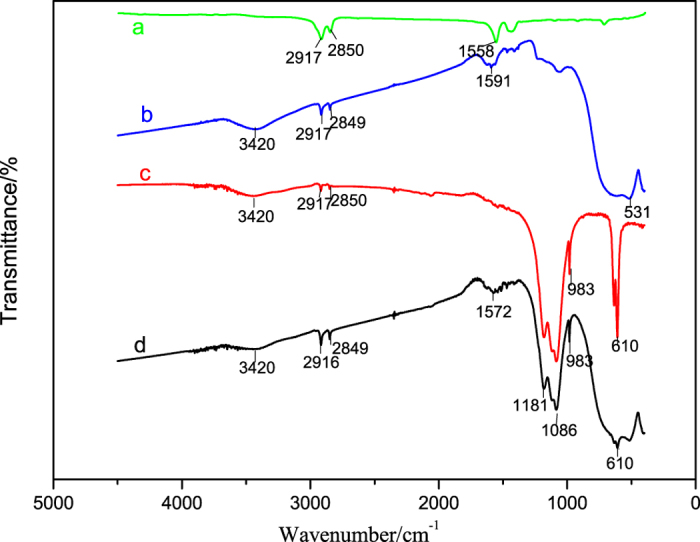
IR spectra of (**a**) sodium stearate, (**b**) the modified TiO_2_, (**c**) the modified barite and (**d**) the TiO_2_-coated barite composite pigments.

FTIR was used to illustrate the characteristic groups of sodium stearate, the modified rutile TiO_2_, the modified barite, and the TiO_2_-coated barite composite pigment (TiO_2_ mass ratio is 60%). The results of FTIR analysis are shown in Fig. 12. In Fig. 12(a), the absorption peaks induced by the stretching vibrations of C-H bonds in the –CH_3_ and –CH_2_- groups appear at 2,917 and 2,850 cm^−1^. Figure 12(b) displays peaks located at below 1,000 cm^−1^, which are induced by the stretching vibrations of Ti-O-Ti bonds. These peaks are a characteristic rutile band^32, 33^. The absorption peak of hydroxyl groups (O-H) appears at 3,420 cm^−1^ because of the intense hydration of unsaturated Ti^4+^. The absorption bands appearing at 2,917 and 2,849 cm^−1^ are induced by the vibrations of C-H bonds^34^. The aforementioned findings are attributed to the adsorption of the sodium stearate group (C_15_H_35_COOH or C_15_H_35_COO^−^) on the surface of the TiO_2_ particles. Because of the existence of the O-H groups in TiO_2_, it can be inferred that sodium stearate was adsorbed on the TiO_2_ particle surface via its reaction with O-H. In Fig. 12 (c), the absorption peaks of -CH_3_ and -CH_2_- at 2,917 and 2,850 cm^−1^ are present, indicating that the modifier was adsorbed on the surface of the barite particles. In addition, the absorption peaks appearing in the range from 900 to 1,200 cm^−1^ are typical SO_4_^2−^ bands^35, 36^ and the absorption bands of a terminal hydroxyl group (O-H) at 3,420 cm^−1^ are induced by the hydration of SO_4_^2^ ^37, 38^. Based on the aforementioned results, sodium stearate was adsorbed on the surface of barite through an adsorption mechanism similar to that of TiO_2_.

In Fig. 12(d), the characteristic adsorption bands of TiO_2_ appear, and there are no new absorption peaks. This observation indicates that the TiO_2_ coated the surface of the barite without chemical bonding. Meanwhile, the absorption bands of -CH_3_ and -CH_2_- also appear with a higher intensity; thus, the composite of TiO_2_ and barite particles is induced by the hydrophobic interaction between the organic carbon chains on their surfaces. Since the interaction can induce the strong intertwining of the extended organic carbon chains on the surface of the particles, the particles are combined firmly with a binding energy that is much stronger than the Van der Waals force^24^.

### Composite mechanism and model

**Figure 13:**
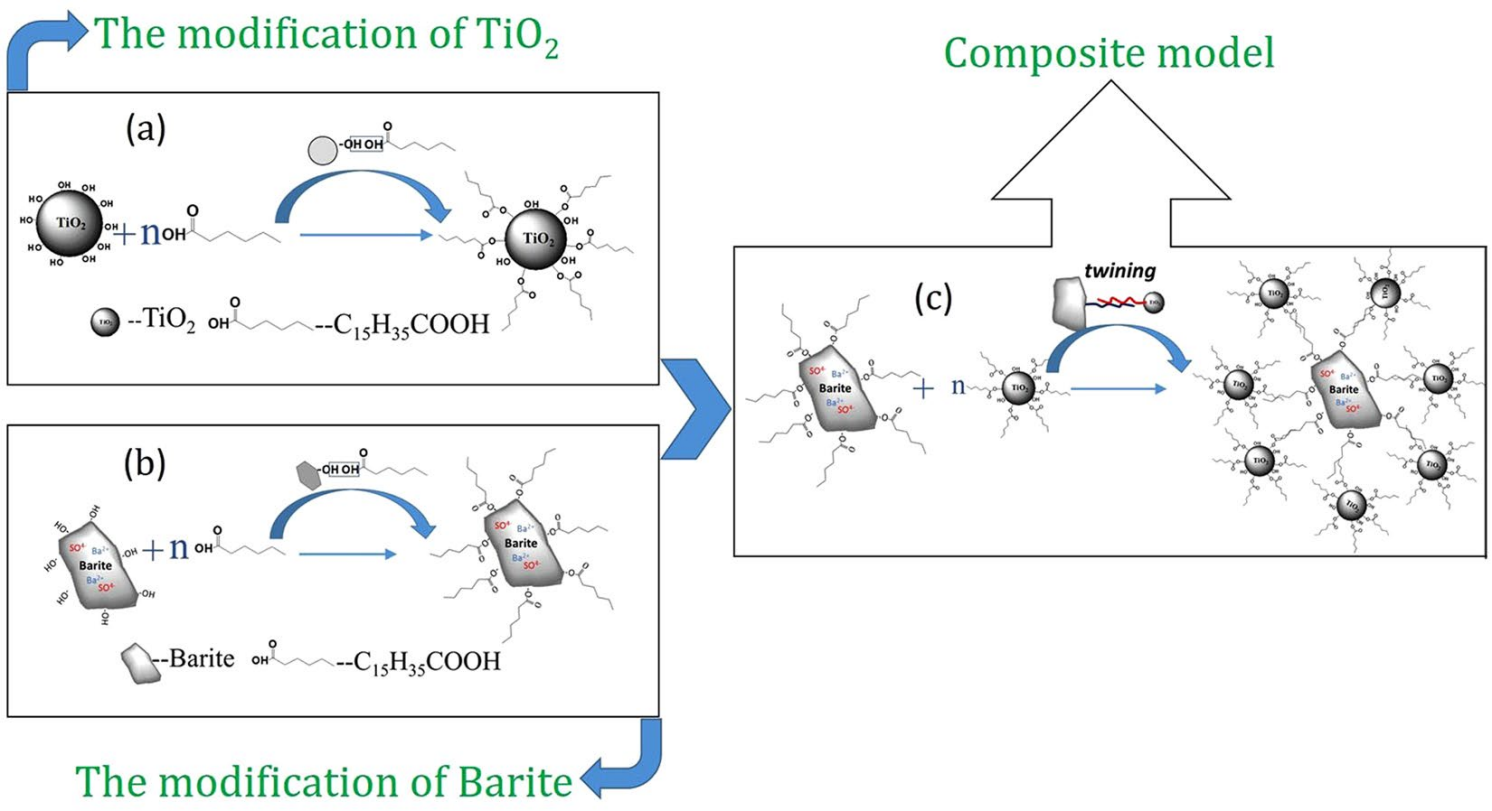
Modification mechanism model of (**a**) rutile TiO_2_, (**b**) barite and (**c**) composite model of the rutile TiO_2_ and barite particles.

Figure 13 shows the composite model of the TiO_2_-coated barite composite pigment. Based on the aforementioned research and analysis, the composite mechanism of the TiO_2_-coated barite composite pigment prepared by hydrophobic aggregation can be described as follows:

First, as Fig. 13(a) and (b) show, the sodium stearate used as a modifier is adsorbed on the barite and TiO_2_ particle surfaces via the reaction between C_15_H_35_COOH and the -OH groups on the surfaces of the TiO_2_ and barite particles. The surfaces of the barite and TiO_2_ particles are then covered by the organic groups of the modifier, and the hydrophobic groups are directed outward, thus increasing the hydrophobicity of particles. Second, the modified TiO_2_ and barite are mixed and stirred in water, and the energy produced by stirring promotes the collision of the particles. Therefore, the distance between particles is reduced to such a range that the carbon chains adsorbed on the surfaces of particles are in contact. Then, the composite of barite and TiO_2_ particles is formed by the interaction of the organic carbon chains (See Fig. 13(c)). Finally, the TiO_2_-coated barite composite pigments with good pigment properties and hydrophobicity are prepared.

Several reasons may account for the good pigment properties and hydrophobicity of the TiO_2_-coated barite composite pigments. Because of the TiO_2_ coating on the surface of barite, the hydration hydroxyl groups on the surface of barite are covered. Meanwhile, the amount of -CH_3_ and -CH_2_- groups increase with the incorporation of the organic carbon chains on the surface of the composite particles.

## Conclusions

Based on the surface hydrophobicity of barite and rutile TiO_2_ particles induced by organic surface modification, rutile TiO_2_-coated barite composite pigments with similar pigment properties to those of rutile TiO_2_ were prepared by heterogeneous particle hydrophobic aggregation in a water medium. When the mass ratio of TiO_2_ was 60%, the hiding power of the composite pigment was 12.08 g/m^2^, which was equivalent to 90.81% of that of pure rutile TiO_2_ pigment. Additionally, the TiO_2_-coated barite composite pigment exhibited a strong surface hydrophobicity and similar optical properties to those of rutile TiO_2_.

The TiO_2_-coated barite composite pigment was characterized as TiO_2_ particles uniformly and compactly coated on the barite particles surfaces. Notably, the coating structure could be formed only when both the surfaces of barite and TiO_2_ particles had strong hydrophobicity. The barite and TiO_2_ particles were combined by the association of the organic carbon chains on their surfaces without chemical reaction.

According to the reaction and bonding characteristics, we established the modification and composite model of the TiO_2_-coated barite composite pigments prepared by the hydrophobic aggregation method.
